# Acute flaccid paralysis secondary to severe hyperkalaemia

**DOI:** 10.1093/omcr/omae023

**Published:** 2025-10-22

**Authors:** Georgina Hadfield, Mark Harrison

**Affiliations:** F2, Northumbria Specialist Emergency Care Hospital, UK; Consultant in Emergency Medicine, Northumbria Specialist Emergency Care Hospital, UK

**Keywords:** hyperkalaemia, flaccid paralysis, emergency medicine

## Abstract

Acute Flaccid paralysis is a rare presentation of severe hyperkalaemia, described rarely in the literature. We report a case of flaccid paralysis, including cranial nerve involvement, secondary to hyperkalaemia in a patient presenting to A&E. Initial ECG showed signs of severe hyperkalaemia and lab results confirmed. The patient was started on hyperkalaemia treatment which reversed his symptoms completely, thus confirming the diagnosis. Flaccid paralysis is a rare presentation of life threatening hyperkalaemia and its recognition is essential in initiating life-saving antihyperkalaemic treatment.

## BACKGROUND

Hyperkalaemia is a life threatening medical condition that can cause ECG changes but is often picked up on blood tests. It can cause acute flaccid paralysis but this may be attributed to other more common diagnoses such as central cord syndrome or Guillain Barre syndrome. It patients presenting with acute flaccid paralysis it is important to consider hyperkalaemia as a possible diagnosis.

## CASE REPORT

We report a case of a 79 year old male who presented to the Emergency Department with a history of worsening mobility over a period of 24 h, which had progressed to complete inability to move his arms or legs. He reported that he had had diarrhoea for the 2 weeks leading up to his admission and had a fall 1 week before his admission.

On examination the patient was fully orientated and alert. He had a Early Warning Score (EWS) of 3 for saturations of 90% on room air, all other observations were within normal range. He was found to have 0/5 power in his proximal limbs with 2/5 power in his toes and 1/5 in his fingers (as per the MRC muscle power scale). All 4 limbs were flaccid with reduced tone and absent deep tendon reflexes. Head and neck were less affected but the patient reported some speech slurring, difficulty swallowing saliva with reduced power in neck and a mild right sided facial droop. His sensation, eye movements and pupillary response were fully intact.

The patient had a past medical history of thrombotic stroke, right bundle branch block and chronic kidney disease. The patient was taking irbesartan and spironolactone for hypertension but had not commenced any new medications and took no non-steroidal anti-inflammatory drugs (NSAIDs). Clinically, there was concern about potential Central Cord syndrome due to the sudden nature of his weakness following a fall. Other possibilities responsible for his presentation included electrolyte abnormalities, for example severe hypercalcaemia which may show Osborn waves and a shortened QT interval on ECG or hyponatraemia, which would not have ECG changes but can present with global weakness. Due to the patient’s history, a stroke was one of the key differentials and a CT head was requested for this, however, the medical team were already alerted to his abnormal blood results as the lab had called with the abnormal potassium therefore he was started on hyperkalaemia treatment.

**Figure 1:**
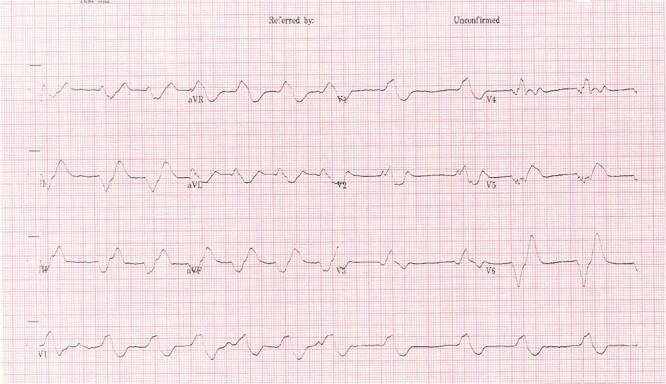
Admission ECG [9].

**Figure 2:**
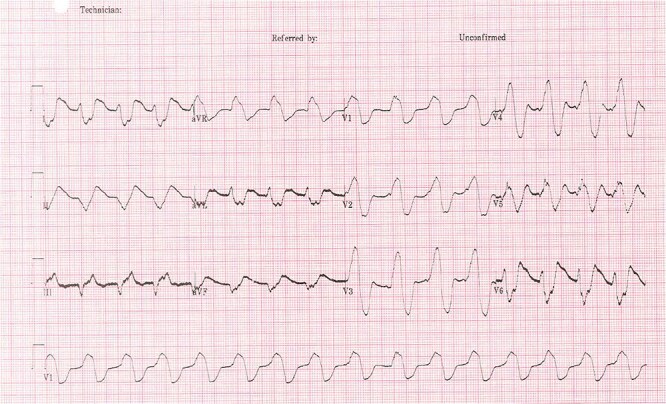
Sinusoidal pattern [9].

The patient’s electrocardiogram (ECG) (Figs 1 and 2) showed broad irregular complexes in a near sinusoidal pattern. Laboratory tests showed a raised urea 31 mmol/l with a slightly raised creatinine 200 mmol/l (baseline 153 mmol/l) and serum potassium of 9.9 mmol/l. An arterial blood gas (ABG) showed he was acidotic at 7.15 with a potassium of 10.5 mmol/l and a BE of −12. During this time the patient was on a cardiac monitor having runs of pulsed ventricular tachycardia and a sinusoidal ECG.

**Figure 3:**
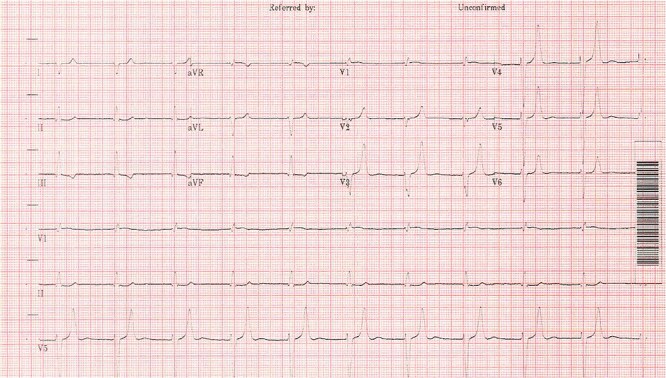
Post treatment ECG, 5 h from admission [9].

The patient was commenced on hyperkalaemia treatment consisting of calcium gluconate, nebulised salbutamol and insulin/glucose infusion. After 45 min (two rounds of insulin/glucose) his speech began to improve and movement began to return, starting with the arm through which the insulin/glucose infusion was running. Within 2 h of treatment his acute flaccid paralysis had fully resolved. Hyperkalaemia treatment was continued, and the patient was transferred to the intensive care unit (ICU) where renal replacement therapy was initiated (Fig. 3). The patient was discharged successfully a week later with a new baseline creatinine of 194 mmol/l. It was concluded that the dehydration from the diarrhoea had led to acute-on-chronic renal failure, with irbesartan and spironolactone exacerbating this problem thus causing the hyperkalaemia. There were no other pre-existing conditions considered likely to contribute to the patient’s hyperkalaemia.

## DISCUSSION

Hyperkalaemia is often an incidental finding, but can present with cardiac, neurological and gastro-intestinal disturbance [1, 2]. Initially, in this case it was overlooked as a differential for the cause of this patient’s paralysis in favour of more common causes such as central cord syndrome or Guillain Barre syndrome.

In our case we noticed a purely motor loss of function of all four limbs without sensory loss, a tendency seen in other cases. Previous case reports have also documented this phenomenon [3–8], yet our case appeared unique in that there was considerable head and neck involvement, which does not seem to be documented in the available literature [3].

In severe hyperkalaemia, the same principle of reduced contractility and conductivity [4], seen in the myocardium, also applies to every muscle in the body including respiratory muscles and limbs causing reversible (acute flaccid paralysis) paralysis [5]. Acute flaccid paralysis is a rare neurological illness often due to inflammation of the spinal cord which can be caused by polio virus, snake venom or Guillain Barre Syndrome [6]. However, this neurology can be mimicked by severe hyperkalaemia and is an important diagnosis to rule out in people with acute flaccid paralysis, especially if there are changes on ECG.

The classic ECG changes seen in this patient alerted clinicians to hyperkalaemia as probable cause of the paralysis. However, this is not a particularly sensitive way to identify hyperkalaemia prior to laboratory results, as studies have shown fewer than half of patients with a high potassium will have ECG changes [7]. Action potentials through neurons are key to initiating muscle contractility and potassium ions (K+) play an important role in changing the membrane potential of cells and thus initiating an action potential. Voltage gated sodium channels open to increase the membrane potential of the cell, initiating the action potential, then voltage gated potassium channels open and K+ ions move out of the cell to repolarise the cell. Where the diffusion gradient of potassium is less, there is impaired repolarisation of the cell resulting in failure of further muscle contraction [4]. When ECG changes occur, it tends to be in a characteristic pattern due to the varying sensitivities of the cardiac tissues to potassium [7].

These signs of Hyperkalaemia seen on the ECG are very important and this bedside test can aid in diagnosis of Hyperkalaemia. However, it should be noted that the sensitivity for picking up hyperkalaemia is poor and lab results should always be used to monitor the severity of the hyperkalaemia [7, 8]. The importance of bloods as an assessment tool in the Emergency Department (ED) should not be forgotten and in resource poor countries where blood results may take longer to be reported the ECG will be invaluable.

### Learning points

- Recognising signs of hyperkalaemia on ECG are very important
- Sensitivity for picking up hyperkalaemia is poor and lab results should be used
- The importance of bloods as an assessment tool in the ED should not be forgotten.

## References

[1] Panichpisal K, Gandhi S, Nugent K, Anziska Y. Acute quadriplegia from hyperkalemia: a case report and literature review. Neurologist 2010;16:390–3. 21150391 10.1097/NRL.0b013e3181b120b8

[2] Habaragamuwa B, Halpegamage N. Flaccid motor paralysis induced by hyperkalaemia. J Neurol Neurophysiol 2012;3:4.

[3] Evers S, Engelien A, Karsch V, Hund M. Secondary hyperkalaemic paralysis. J Neurol Neurosurg Psychiatry 1998;64:249–52. 9489541 10.1136/jnnp.64.2.249 PMC2169962

[4] Weiss J, Qu Z, Shivkumar K. Electrophysiology of hypokalemia and hyperkalemia. Circ Arrhythm Electrophysiol 2017;10:e004667. 28314851 10.1161/CIRCEP.116.004667 PMC5399982

[5] Hemachandra K, Dayasiri M, Kannangarra T. Acute ascending flaccid paralysis secondary to multiple trigger factor induced hyperkalaemia. Case Rep Neurol Med 2018;2018:1–4. 10.1155/2018/6360381 PMC5996444 30002937

[6] Naik K, Saroja A, Khanpet M. Reversible electrophysiological abnormalities in acute secondary hyperkalaemic paralysis. Ann Indian Acad Neurol 2012;15:339–43. 23349611 10.4103/0972-2327.104354 PMC3548384

[7] Montague B, Ouellette J, Buller G. Retrospective review of the ECG changes in hyperkalaemia. Clin J Social Nephrol 2008;3:324–30. 10.2215/CJN.04611007 PMC2390954 18235147

[8] Udezue E, Harrold B. Hyperkalaemic paralysis due to spironolactone. Postgrad Med J 1980;56:254–5. 7433326 10.1136/pgmj.56.654.254 PMC2425886

[9] Electrocardiograms obtained from Patient’s notes, written permission obtained from him for these images to be used.

